# Achieving Maximal Speed of Solution Exchange for Patch Clamp Experiments

**DOI:** 10.1371/journal.pone.0042275

**Published:** 2012-08-03

**Authors:** Jerónimo Auzmendi, Darío Fernández Do Porto, Carla Pallavicini, Luciano Moffatt

**Affiliations:** Instituto de Química Física de los Materiales Medio Ambiente y Energía, Facultad de Ciencias Exactas y Naturales, Universidad de Buenos Aires, Buenos Aires, Argentina; Monell Chemical Senses Center, United States of America

## Abstract

**Background:**

Resolving the kinetics of agonist binding events separately from the subsequent channel gating processes requires the ability of applying and removing the agonist before channel gating occurs. No reported system has yet achieved pulses shorter than 100 µs, necessary to study nicotinic ACh receptor or AMPA receptor activation.

**Methodology/Principal Findings:**

Solution exchange systems deliver short agonist pulses by moving a sharp interface between a control and an experimental solution across a channel preparation. We achieved shorter pulses by means of an exchange system that combines a faster flow velocity, narrower partition between the two streams, and increased velocity and bandwidth of the movement of the interface. The measured response of the entire system was fed back to optimize the voltage signal applied to the piezoelectric actuator overcoming the spurious oscillations arising from the mechanical resonances when a high bandwidth driving function was applied. Optimization was accomplished by analyzing the transfer function of the solution exchange system. When driven by optimized command pulses the enhanced system provided pulses lasting 26 ± 1 µs and exchanging 93 ± 1% of the solution, as measured in the open tip of a patch pipette.

**Conclusions/Significance:**

Pulses of this duration open the experimental study of the molecular events that occur between the agonist binding and the opening of the channel.

## Introduction

The kinetic study of ligand-gated ion channels depends on the ability to rapidly exchange agonist concentration on outside out patches. This ability is of great importance in different fields. In Ligand Gated Ion Channels (LGIC) biophysics the application of very short pulses of the agonist [1] is useful to resolve pre-opening conformational changes [2], [3] related to the mechanisms of activation [4], [5] and to solve the problem of separating binding and gating steps [5], [6]. In synaptic neurophysiology, the ability to experimentally mimic the release of the neurotransmitters is key to understanding the role played by these channels in information processing [7]. In pharmacology of LGIC and other ion channels, the analysis of the kinetic response to short pulses of drugs helps to elucidate the mechanism of action of the pharmacological agents [8]–[10].

Methods to exchange the solution to which an ion channel is exposed include photo-release and hydraulic techniques [11], [12]. Photo-release requires elaborate synthesis of special compounds. A drawback of photo-release techniques is the difficulty in knowing the actual concentration of the compound at the patch location. Furthermore agonist removal relies in diffusion, limiting the clearance time [13], [14]. Hydraulic techniques are based on the rapid movement of a sharp interface between a pair of streams positioned very close together. The movement of the interface can be driven by either switching off one of a pair of colliding streams with the aid of a valve [1], [11], [15] or by translating both parallel streams in a direction perpendicular to the flow with the aid of a piezo actuator [15], [16]. Hydraulic exchangers achieve sub millisecond rise time, close to that for the photo-release delivery systems, while maintaining the same time resolution for the removal of the drug and knowing the actual concentration at the patch location.

In the present state of the art, the generation of agonist pulses achieves durations of 200–400 µs [3], [17]. Commercially available devices generate pulses of 1 ms. In order to study the activation of Ach receptors [18]–[23] and AMPA receptors [13], [14] which have a gating constant of 20–100 µs much shorter pulses are needed. Ideally the application of the agonist should occur on a timescale on the order of 20–50 µs. A theoretical study using finite element models of a dual stream switcher [24] suggests that it should be possible to achieve such short pulses. The aim of this paper was to experimentally produce 20 µs pulses in an open tip configuration.

The primary factors that limit the speed of solution exchangers have been discussed by Sachs [24]. The flow velocity, the width of the partition between the two streams and the speed at which current can move through the patch are the key parameters. We optimized each one of these factors to obtain the shortest pulses possible. The optimized solution switching system is able to achieve 90% of solution exchange in pulses briefer than 30 µs.

**Figure 1:**
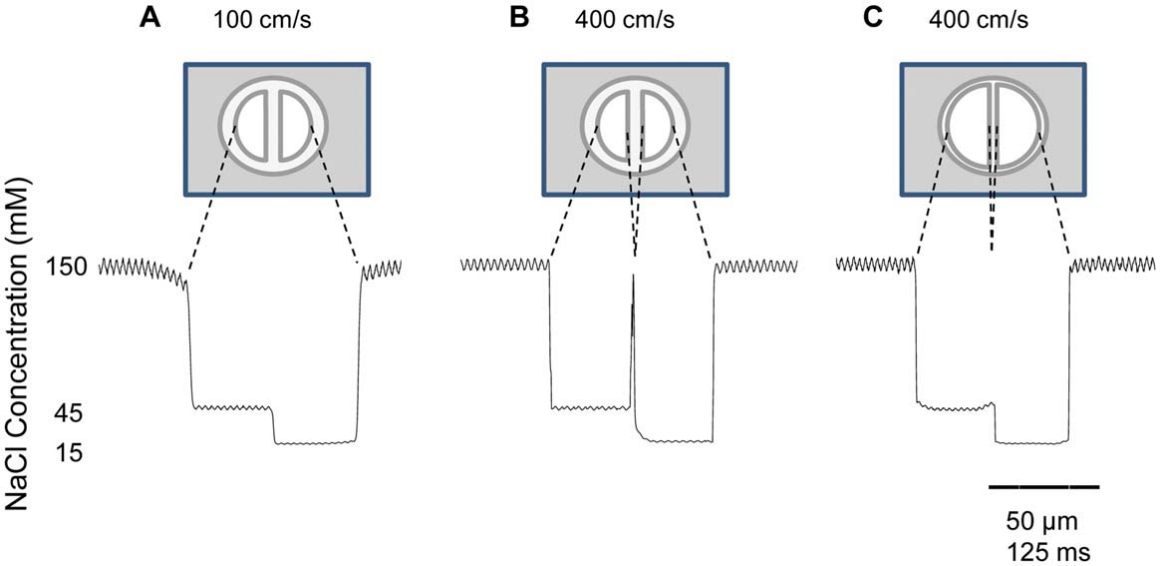
Effects of flow velocity and septum width on the transverse profile of the concentration. The traces show the actual current measured at −100 mV on a recording pipette with an open tip moving at a constant velocity of 0.4 mm/s. (*A–B*) Concentration profile for an untreated application pipette with a septum width of 10 m at 100 cm/s and 400 cm/s respectively. In the later case, a spike at the boundary between channels indicates that solution from the bath is being dragged into the interface between streams. (*C*) Concentration profile for an application pipette with the septum thinned to 3 µm with the measures at 400 cm/s flow velocity. The concentration profile is comparable to that in A.

## Materials and Methods

### Experimental Device, Perfusion System & Solutions Exchanger

All experiments were done on a patch clamp setup adapted for ultra fast pulses. High solution flow rates were obtained by means of a pressurized system composed of a small nitrogen cylinder (10 l) as a constant pressure source. To avoid oscillation in the flow rate, pressure regulators were not used. Indeed, as the nitrogen consumption was relatively small (70 ml/h), the pressure was constant during the course of an experiment. A homebuilt pneumatic system was used to drive the syringes containing the solutions. This arrangement was made of two 60 ml syringes (Neojet, Propato, Buenos Aires, Argentina) joined back to back via their plungers. Thus, one set was connected to the pressurized nitrogen; the other set was filled with experimental solutions and connected to the application pipette. The first set of syringes worked as pneumatic cylinders pushing the plunger of the second syringes set, thereby avoiding the contact of the pressurized nitrogen with the solutions. This prevented the dissolution of the nitrogen into the exchanged solution which leads to the formation of bubbles at the patch. The syringes delivered the solution through polyethylene tubing (PE-50, Warner Instruments, Hamden, CT) to the application pipette. The latter was mounted on a metal holder glued with epoxy glue (Poxipol, Acapol, Buenos Aires, Argentina) to one extreme of a piezoelectric actuator stack (AE0505, Thorlabs, Newton, NJ). The piezoelectric stack was epoxy-glued to an acrylic base and supported on a 3D translation stage (461-XYZ-M, Newport, Irvine, CA). A custom made piezo amplifier built around a 50 kHz, 30 A power operational amplifier (PAD115, PowerAmp Design, Tucson AR) was used to drive the piezo stack. A multifunction data acquisition (NI USB 6259, National Instruments, Austin, TX) was used to drive the piezo amplifier as well as for acquisition of the patch clamp amplifier data. The recording pipette headstage was mounted on a motorized 3 dimension manipulator (860, Newport) and connected to a patch amplifier (2400, A-M Systems, Sequim, WA). The frequency response of the patch clamp amplifier was tested with a capacitor circuit element and its transfer function was obtained. A nearly ideal capacitor was built by bringing two conductors close to each other [25], one conductor was the patch pipette, the other a stripped wire connected to the analog output of the data acquisition card. The response to a triangle wave was measured, since the coupling capacitance carries a current that is the derivative of the applied voltage, and the derivative of a triangle wave is a square wave. We also measured the response to a square wave to derive the transfer function of the amplifier. The bandwidth of the patch clamp amplifier was adjusted to 80 KHz by following the instructions of the manufacturer. Data were digitized and recorded at 500 kHz.

**Figure 2:**
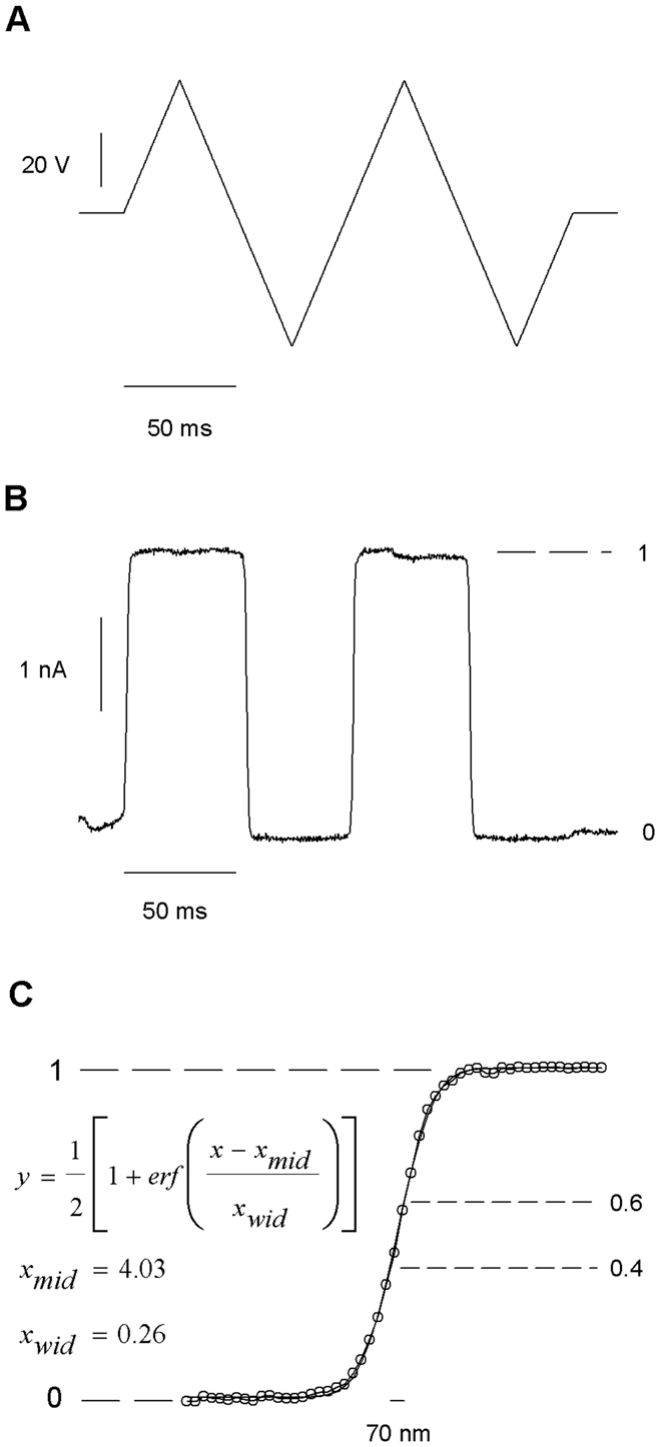
Interface between streams. The measures were made with a recording pipette still while moving the application pipette with the piezoelectric actuator. (*A*) Triangle shaped driving voltage causing the recording pipette alternate between the two solution streams. (*B*) Recorded current profiles at −100 mV after crossing between external solution and external solution diluted 1∶10. (*C*) Higher resolution current profile for the coordinates used in B. The width of the interface was estimated by assuming a linear movement of the piezo actuator of 0.1 µm/V. The broken lines indicate a region were the changes in current are approximately proportional to the changes in position. The solid line indicates a fit to the plotted equation.

A pressure of 1–1.2 bar was applied to the pistons to generate a flow rate of 15–20 µl/s of solution per line. As the diameter of the application pipettes varied in the 100–120 µm range, the flow rate was adjusted accordingly for the area of the tip of the application pipette to assure that the average velocity of the fluid would be ca. 400 cm/s at the tip of the application pipette. The calculation of the flow velocity was made assuming that, at a distance that is half the radius of the application pipette tip, the flow stream diameter equals that of the application pipette tip.

**Figure 3:**
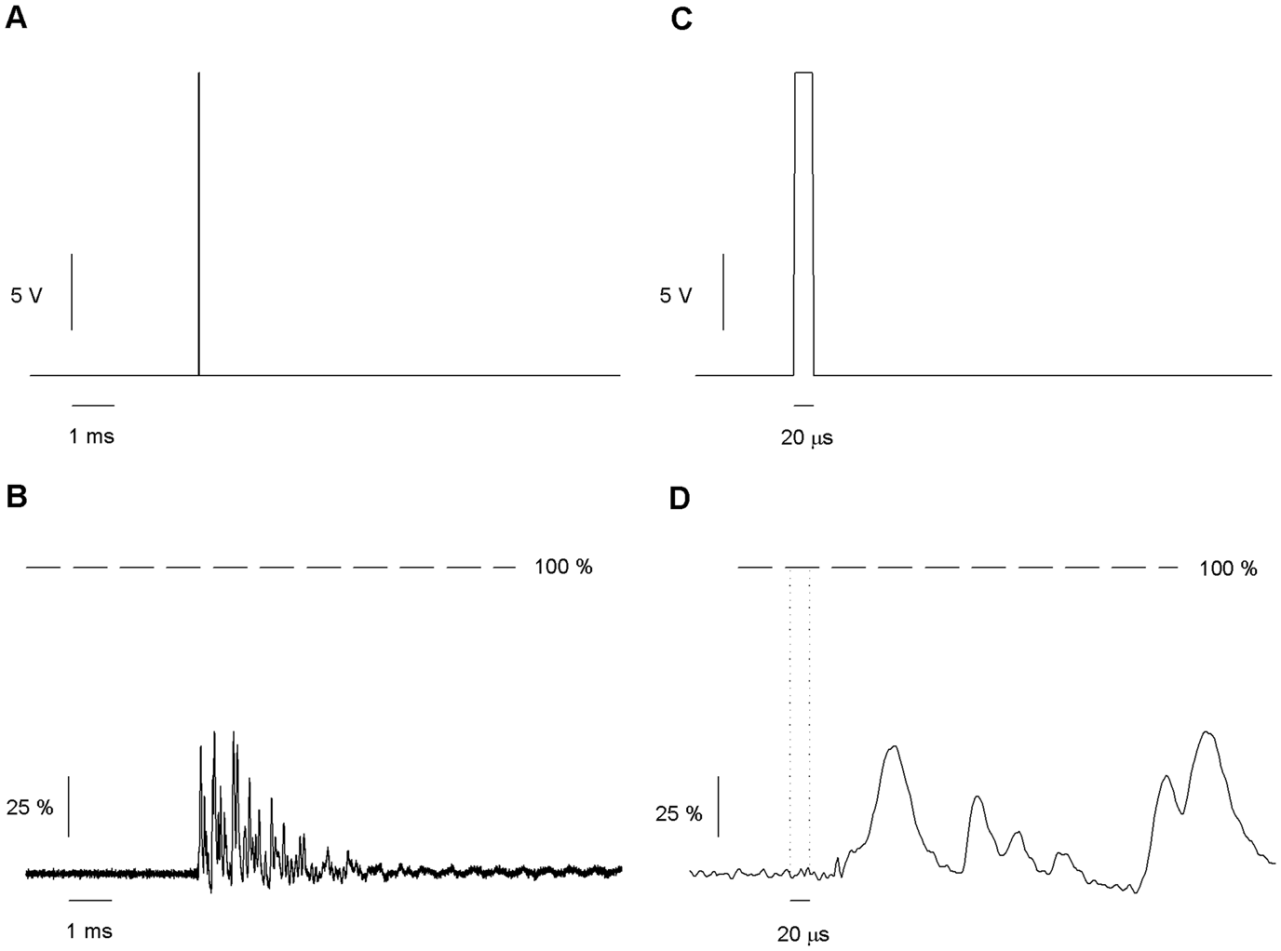
Response of the solution exchanger to a short pulse. (*A*) A 20V 20 µs square pulse applied to the piezoelectric actuator at 1 Hz of repetition rate. The recording pipette is positioned at 0.85 µm from the interface. (*B*) Changes in current generated by the movement in response to the pulse. Dashed line indicates the current expected for a complete solution exchange. Mechanical oscillations of the piezoelectric translated into repeated partial solution exchanges. (*C–D*) Expanded scale of A and B. (*D*) Show the onset of the motion. The dotted line marks the position of square voltage command.

The application pipettes were made of a borosilicate theta tubes (BT 150-10, Sutter Instruments, Novato, CA) pulled on a horizontal micropipette puller (P-97, Sutter Instruments); the tip diameters of the application pipettes were adjusted down to 100–120 µm of outer diameter by carefully hand-grinding the tips with diamond lapping films of 1 µm (LFGxD, Thorlabs, Newton, NJ). After grinding, the pipettes were cleaned 1 h in a solution of 0.1 M KMnO_4,_ 2.5 mM NaOH and washed with a solution of 7.5% H_2_O_2_ 2.5 mM H_2_SO_4_. To reduce the width of septum wall of the theta tubes from 10 to 3 µm the pipette tip, was filled up to 1–2 mm with 10% HF [26] in absolute ethanol for 20 min and then washed 3 times with bi distilled water. A piece of Ultramicrobore PTFE tubing of 0.2 mm ID, 0.4 mm OD (EW06417-76, Cole-Parmer Instrument Co, Vernon Hills, IL) was inserted 10 mm into each line of the application pipette and sealed with epoxy. The success rate for pipette fabrication can be as high as 90%. The application pipettes just made were stored by dipping the tip in a solution of 1 M HClO_4_ when finished. All experiments were performed using recording pipettes (GC150F-7.5, Harvard Apparatus, Holliston, MA) pulled in the micropipette horizontal puller to a resistance of 10–15 MΩ when filled with an internal solution containing 145 mM CsF, 5 mM NaCl, 1.3 mM MgCl, 10 mM HEPES. As 100%, 30% and 10% external solution we used 150, 45 and 15 mM NaCl respectively; but with the same concentration of HEPES, 10 mM. The pH of all solutions was adjusted to 7.4 with aqueous HCl. The distance between recording and application pipettes was set at half the radius (*ca*. 25 µm) of the application pipette and the angle between them was set at 105°.

**Figure 4:**
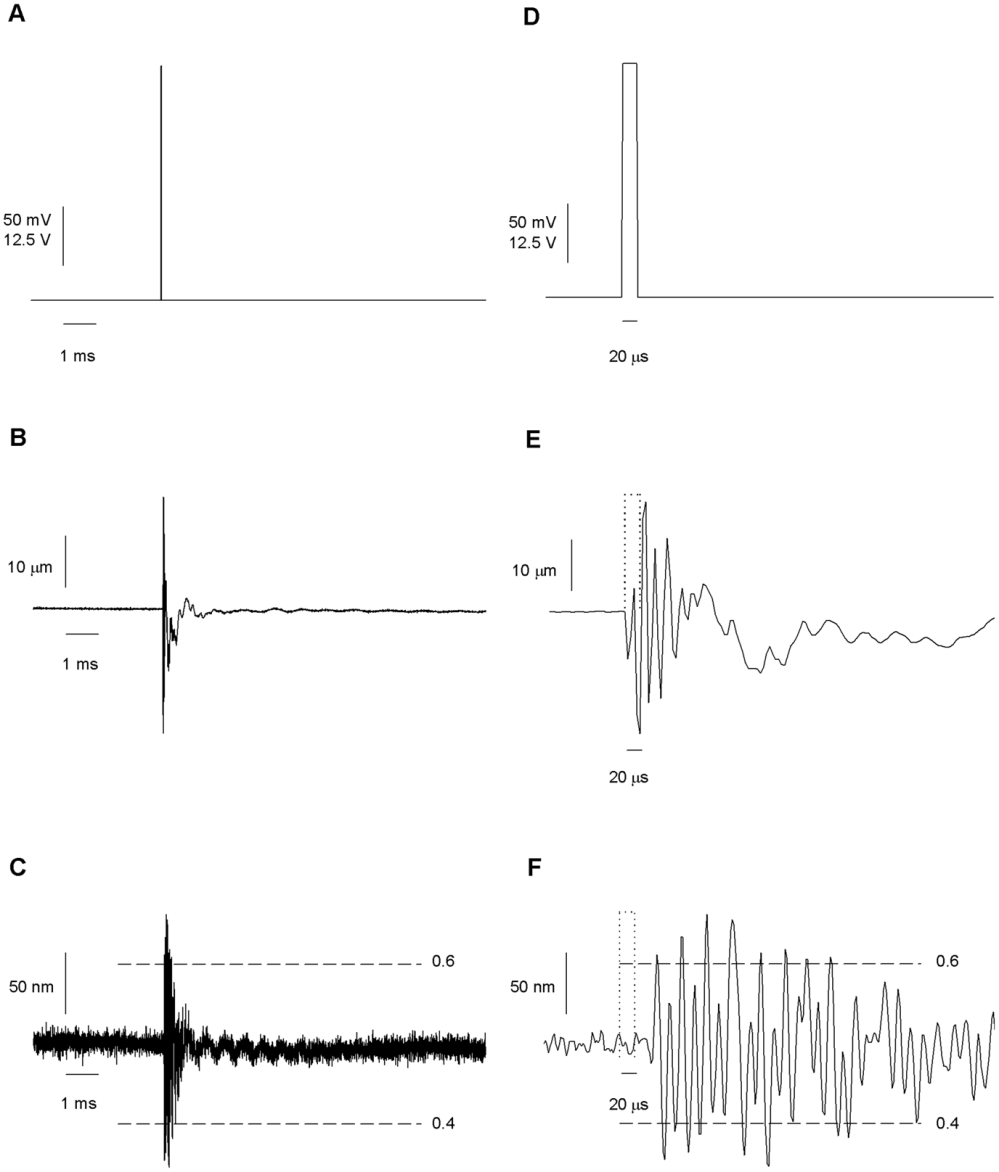
Movements of the piezo and the interface after a short pulse command. The measures were made optically for the piezo motion and with the recording pipette positioned at the center of the interface. (*A*) Big amplitude (50V) or a small amplitude (200 mV) 20 µs command square voltages were applied to the piezo. Big amplitude pulses generate movements big enough to be properly measured. Small amplitude pulses confine the movement of the interface to the linear displacement region. (B) Piezo motion in response to a big pulse. (C) Interface movement in response to voltage command. Calibration of the movement was done after data of figure 2C (*D*) Expanded scale of A. (E–F) Higher resolution views of the movement pattern generated by the pulsed activation of the piezo. Dotted line indicates the time of the pulse command. 100 and 148 traces were averaged for piezo and interface movements respectively.

### Black Box Approach

The transfer function of the response of a piezoelectric stack alone has been previously measured to optimize the movement of the piezo [27]. Here we extend this idea to optimize the movement of the whole system that is constituted by the piezo stack + manipulator + application pipette + liquid interface. To calculate the system response, we measure the movement of the interface after a very short (20 µs) and very small (200 mV) pulse applied to the piezo. The movement of the interface is registered by the changes it produces in the open tip current of a patch pipette positioned at the middle of the interface. As a first order approximation we assume that the movement of this system can be described by a linear system of differential equations. This is equivalent to consider the whole solution switching system as a single unknown Black Box, which is completely determined by its Transfer Function G(ω), the ratio between the Fourier transform of the measured movement of the solution interface y_m_(*t*), and the Fourier transform of the voltage command applied to the piezo v_c_(*t*):


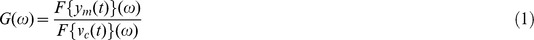


Where F {f(*t*)}(ω) indicates the Fourier transform of the function f(*t*). The voltage command v_d_(t) that should generate the desired displacement y_d_(*t*) is then:


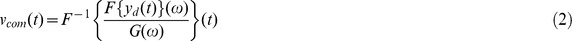


Here F^−1^ indicates the inverse Fourier transform. Because the measurements were finite in time and discrete in sampling, the actual Fourier transform was not used. We used instead the fast Fourier Transform (FFT) algorithm to obtain the discrete Fourier transform and it’s inverse. We also used the Black Box approach to correct the transfer function of the patch clamp amplifier and get a better estimate of the rise and fall times of the pulses.

**Figure 5:**
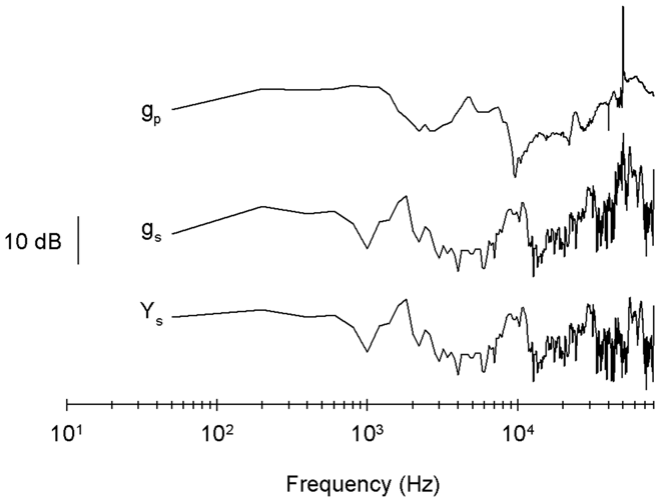
Power spectrum and the transfer function of the movement of the piezo alone and of the interface. Power spectrum (Y_s_) and the corresponding transfer function of movement of the piezo alone (g_p_) and of the interface (g_s_) calculated from the data in Figure 4.

### Response of the Piezoelectric Stack: Optical Measurements

Piezo motion upon a voltage pulsed command was measured by the displacement of a laser beam reflected by a gold mirror attached to one end of the piezo. The angle of incidence of the beam was 45°; the movement of the beam was measured with a quadrant photodiode detector placed 6 cm from the incidence point. The reading of the quadrant photodiode was calibrated after moving the piezo known distances with the motorized manipulator (860 Newport).

**Figure 6:**
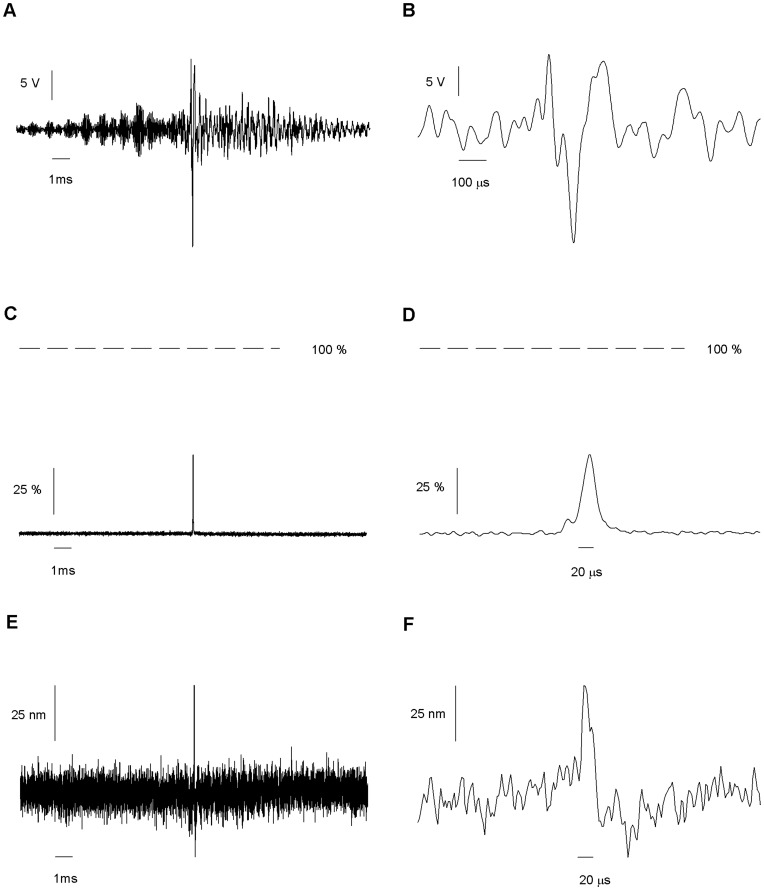
Response to a voltage command optimized for a pulsed movement. (*A*) Shape of the optimal command, calculated on the basis of the response depicted in Figure 4B to obtain a 20 µs pulse. (*B*) Expanded scale of A. (*C*) Measurement of the solution exchange, expressed as percentage of a complete exchange, generated by the application of optimized command scaled to have a maximum excursion of 20 V. A clean but incomplete exchange is clear. (*D*) Expanded scale of C. (*E*) Measurement of the movement of the interface after the application of the optimal command, but scaled to have a maximum excursion of 250 mV. (*F*) Expanded scale of E. The measurements were the average of 100–120 traces.

## Results

### Characterizing the Interface

In order to reduce the rise time of the solution exchanger, we varied the pressure applied to the syringe pistons in such a way that the estimated flow velocity out of the application pipette changed between 100 cm/s and 400 cm/s. We measured the current on the recording pipette as we activated the motorized manipulator (velocity: 0.4 mm/s) to move the recording pipette from one stream to the other. We found that the drop in current that occurs when crossing the interface between the control solution (external solution) and the experimental solution (10% external solution, 90% water) showed the expected shape of a diffusive process (i.e., one described by an error function) at 100 cm/s. However, after we increase the velocity of the fluid to 400 cm/s, the interface showed a staircase shape. We hypothesize that fluid from the bath is being drawn into the interfacial region between the two flows. To test that possibility we set a higher concentration of external solution in the surrounding bath than in the ports: 30% external solution on one port, 10% external solution on the other, while the bath was constantly replenished with 100% external solution (Figure 1). In this way, the presence at the interface of current values corresponding to concentrations higher than that of either port would prove our hypothesis. At 100 cm/s the change in the current at the interface was monotonic thus suggesting that the contamination was negligible at this velocity (Figure 1A). When the flow velocity was increased to 400 cm/s the current at the recording pipette increased to values corresponding to the concentration of the bath showing that the interface between streams was contaminated with bath solution. Therefore, in this situation there were two interfaces: one between the 10% external stream and the dragged bath solution and another one between the latter and the 30% external stream (Figure 1B). The distance between the two interfaces, estimated assuming a constant velocity of the motorized stage was 2.8 µm, representing a 27.5% of the septum width. These results are consistent with the streams being more parallel at greater flow velocities.

**Figure 7:**
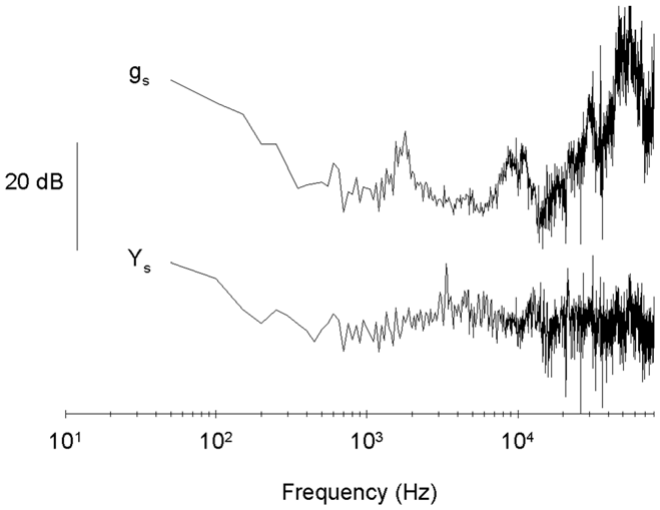
Power spectrum and the transfer function of movement. Power spectrum (Y_s_) and the corresponding transfer function (g_s_) of movement of the interface calculated from the data in Figure 6.

It was found that a smaller septum counteracts the effect of increasing the velocity very efficiently. Etching the application pipette with hydrofluoric acid 10% in absolute ethanol allowed reducing the width of the septum from 10 to 3 micrometers. Reducing the width of the septum allowed us to maintain an ideal profile and maintain a fluid velocity of 400 cm/s (Figure 1B and C).

**Figure 8:**
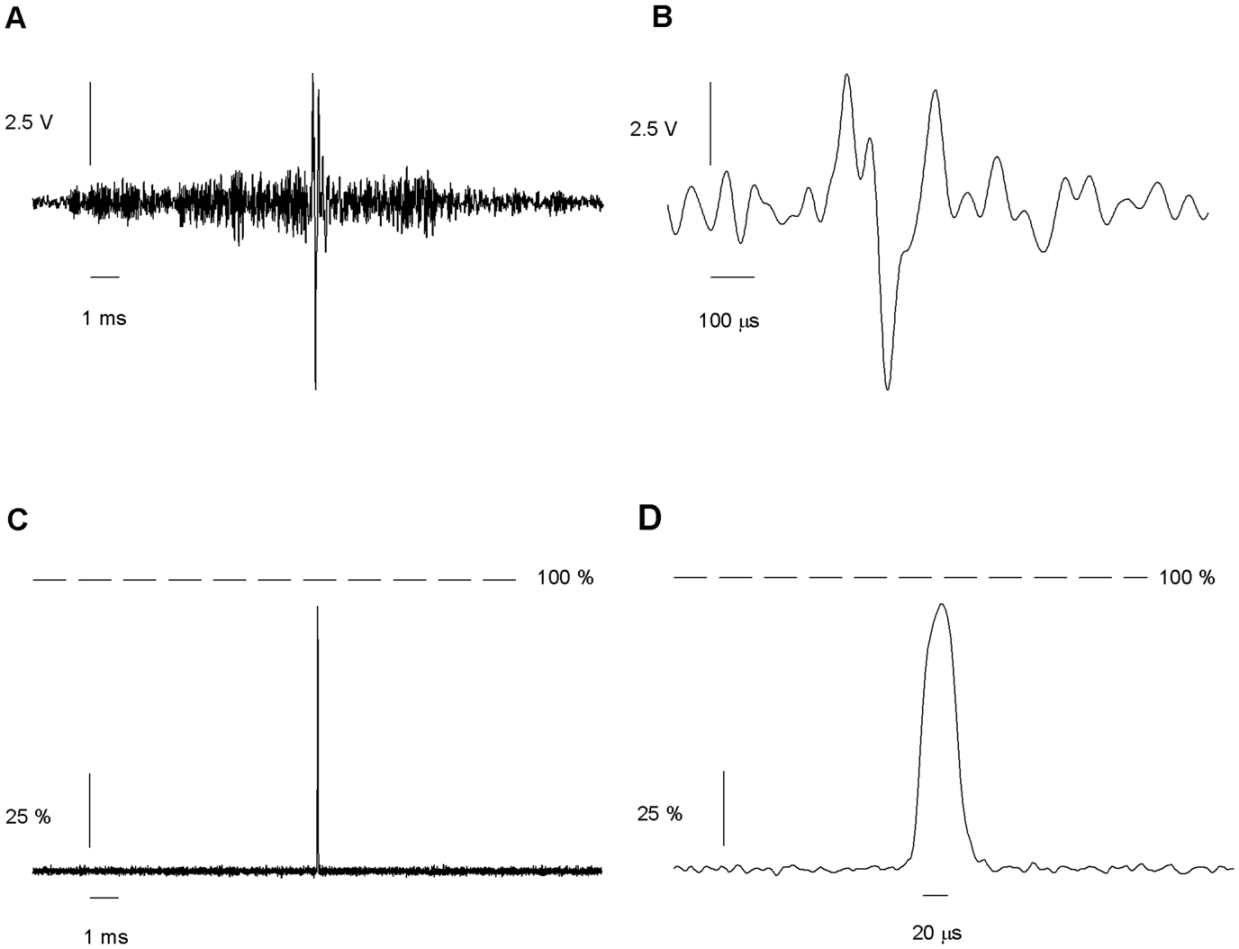
Response to a further optimized command. (*A*) Shape of the optimal command calculated on the basis on the transfer function depicted in Figure 7. (*B*) Expanded scale of A. (*C*) Measure of the solution exchange after the application of the optimal command, expressed as percentage of a complete exchange. A single pulse that crosses almost the entire interface was obtained by applying the optimal command with a maximum excursion of 10 V (*D*) Expanded scale of C. The width of the pulse is about 25 µs. Average of 150 traces.

The next step was to accurately measure the interface between both streams of solution. In the following experiments we use only two solutions, 100% external at one stream, 10% external at the other stream, to increase the signal to noise ratio, critical at high frequency recordings. For the experiments in Fig 1 the recording pipette was moved, while for the remaining experiments it is the application pipette that is being moved. From the point of view of channel kinetics, the parameter of interest is the concentration profile of the agonist as we travel from one stream to the other. However, we measured the changes in the patch pipette current while holding the potential at −100 mV. Since the expected junction potential is only about 14 mV, only a small part of the changes in current (less than 14%) depart from a linear relationship with the changes in concentration. To characterize the interface we applied a triangle wave to the piezoelectric actuator (Figure 2A) moving the interface in such a way that the patch pipette tip alternated between both streams. We found that the current remained constant within the streams, changing abruptly at the interface. The 10–90% range of the concentration profile span a distance of 0.36 µm. The concentration profile at the interface is well described by an error function. The 40–60% region near the inflection point is approximately linear (Figure 2B and C). Therefore, positioning the recording pipette in this range yields changes in current proportional to the displacement of the interface between the streams.

**Figure 9:**
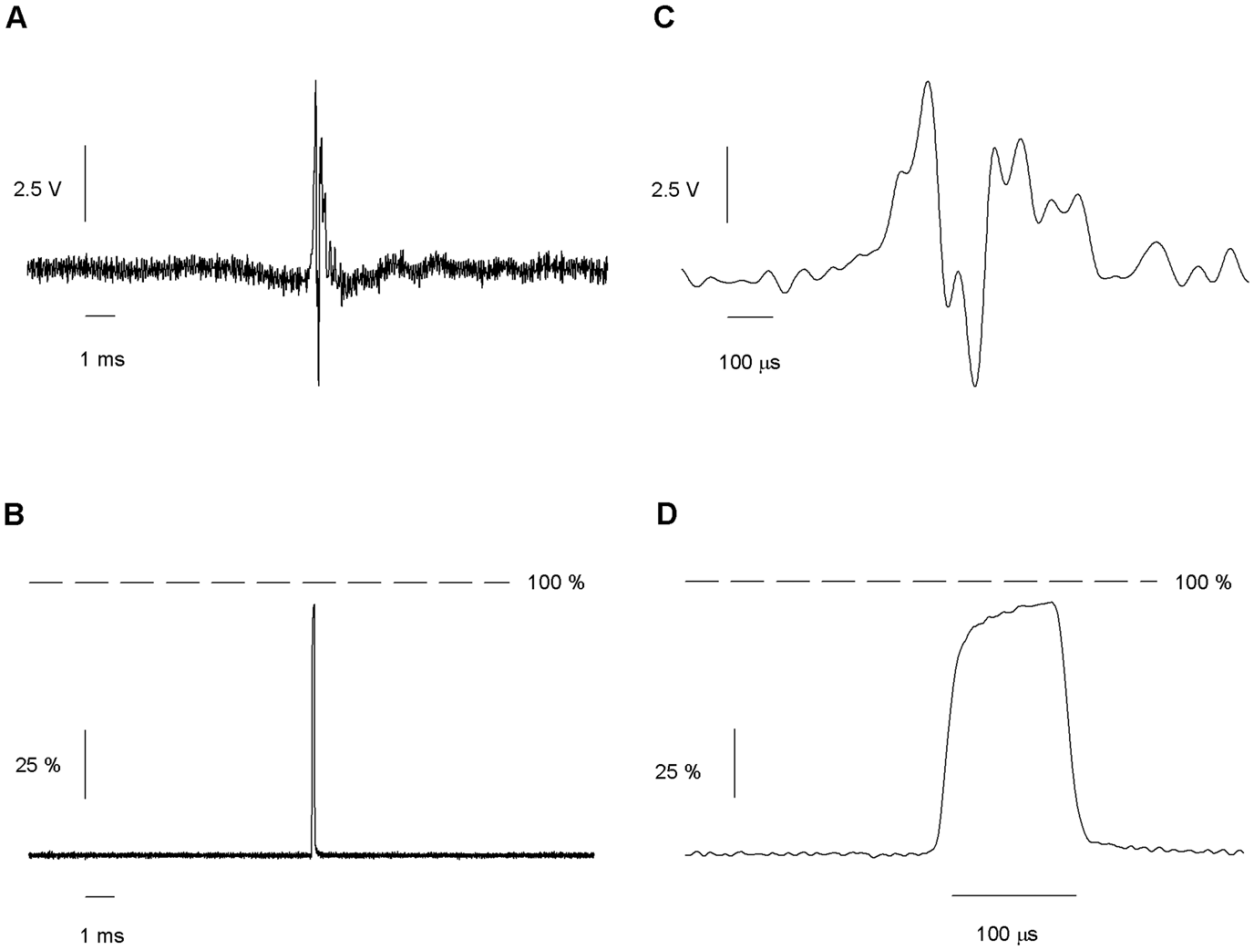
Response to the optimized command for pulses of 100 µs. (*A*) Optimal command calculated after two cycles. (*B*) The same graph with a larger scale. (*C*) Solution exchange shows a single pulse after amplitude of 10 V. A larger scale is shown in D. The mean duration of the pulse is approximately 100 microseconds. Average of 130 traces.

### Response to a Simple Square Pulse

Taking advantage of the information present in Figure 2, we positioned the measuring pipette at 0.85 ± 0.05 µm of the interface and we applied a square pulse (amplitude: 20 V, duration: 20 µs) to the piezo and recorded the patch pipette current. The average response of 148 traces is shown in Figure 3A. To ensure that the interface did not drift away from the pipette its location was verified every 10 applied pulses. This was accomplished by modulating the stream interface position by means of a triangular waveform applied to the piezo while monitoring the pipette current (Figure 2A and B). The averaged response showed that the 20 µs pulse induced a complex current response having multiple peaks, none of which reached 100% of solution exchange. This suggested that the interface repeatedly came close to the position of the recording pipette but never completely surpassed that position (Figure 3B). The movements of the interface lasted several milliseconds and there was a delay of 50 µs between the command voltage pulse and the motion of the interface (Fig. 3C and D).

The movement of the piezo alone after a 50 V, 20 µs pulse was optically measured (Figure 4B and E). The pulse induced a complex, oscillating motion of the piezo with peak to peak amplitude of 45 µm (0.9 µm/V) which completely damped after 10 ms (Figure 4). The first of four fast oscillations was completed in 18 µs. The piezo started to move no later than 2 µs after the voltage pulse is applied.

By positioning the pipette near the interface, only the movements of the interface occurring close to the application pipette are detected; the movements occurring far from it are missed. On the other hand, by placing the measuring pipette in the middle of the interface and reducing the amplitude of the pulse, it was possible to make a complete measurement of the movement of the interface. The interface was re-calibrated every 10 pulses by applying a triangle signal. We constantly monitored the position of the interface making a fine correction of the position of piezo by varying the holding potential in 0.5 mV steps. This allowed keeping the position of the interface at the beginning of each pulse between 40 and 60% of the difference in current at both sides of the interface. Only traces where the initial position of the interface laid between 40 and 60% were included in the average (see Figure 2C). Figure 4C and F show the average movement induced by a 20 µs 200 mV square voltage pulse. 148 pulses were averaged. The pulse induced a complex oscillating movement of the interface with peak to peak amplitude of 200 nm (1 µm/V) which completely damped after 10 ms (Figure 4). The first of 20 fast oscillations was completed in 18 microseconds. A 42 µs delay was observed before the interface started to move. This lag arises from the 3 cm arm length of the application pipette and the 25 µm distance between the application pipette and the recording pipette.

### Estimating the Transfer Function of the Solution Exchanger

The solution switching system can be modeled as a linear system: the input is given by the voltage fed to the piezo, x(t), and the output is the movement of the interface. The transfer function of this system has enough information to calculate the command voltage needed for the piezo to generate any desired movement within the bandwidth of the electro-mechanical system.

The amplitude of the transfer function of the system, g_s_(ω), and of the piezo alone, g_p_(ω), and that for the Fourier transform of the movement of the system, Y(*ω*) are shown in Figure 5. As the amplitude of the Fourier transform of a square pulse input, X(ω) is mostly flat at frequencies lower than the pulse duration, both Y(ω) and g_s_ (ω) show the same frequency dependence to the up to ca. 50 kHz, where the amplitude of X(ω) drops to zero. The apparent peak of g_s_ (ω) and g_p_(ω) at exactly 50 kHz is an artifact caused by the zero value of X at that frequency. The transfer function of the piezo alone showed multiple resonances (0.97 kHz, 4.54 kHz, 23.9 kHz, 43.6 kHz, 48.1 kHz, 60.4 kHz), that where different from the resonances of the whole system (1.76 kHz, 2.39 kHz, 8.8 kHz, 10.8 kHz, 29.4 kHz, 31.3 kHz, 44.6 kHz, 45.4 kHz, 51.0 kHz, 55.3 kHz, 65.6 kHz).

The optimized command voltage to the piezo was a complex signal that started 10 ms before the time of the intended pulse (Figure 6A) peaked close to the intended time of the pulse (Fig. 6B) and remained 11 ms after the end of the intended pulse. The early portion of the synthesized command compensates for the system lag time and the late portion counteracts the resonant oscillations. To measure the solution exchange that results from using this optimized command pulse we change the baseline voltage of the piezo to position the interface again at 0.85 µm of the measuring pipette and drove the piezo with maximum amplitude of 10 V. As expected the achieved solution exchange was essentially free of spurious oscillations although it only achieved 40% of solution exchange, bellow our target of a 90% exchange. Therefore, we measured the movement of the interface with the aim of measuring the transfer function with greater precision. The movement of the interface was close, but not equal, to the intended squared pulsed shape (Figure 6E). The maximum velocity of movement was 0.4 cm/s at command amplitude of 250 mV. An estimate of the maximum velocity of 16 cm/s achieved at 10 V of command amplitude was obtained by linear extrapolation.

### Further Optimization and a Cleaner Solution Exchange

With the aim of optimize further the movement and obtaining a cleaner solution exchange we calculated again the Fourier transform of the movement of the interface, Y(ω), and the corresponding transfer function G(ω) (Figure 7). Y(ω) was almost flat. G(ω), although similar to its previous estimation, was different enough to suggest the possibility that using it we could get a cleaner solution exchange. We expected this because, in the previous estimate of the transfer function, the response to some frequencies was too small to be measured adequately. We calculated the necessary driving pulse by means of Eq. 2 to generate a 20 µs square liquid exchange pulse (Figure 8). The application of this optimized command signal resulted in a pulse of 26 ±1 µs duration with an exchange amplitude of 93 ± 1 %. The standard deviation of the pulse duration and the exchanged amplitude measured at successive trials were 1.9 µs and 3.2%. The rise time, after correcting for the transfer function of the patch clamp amplifier, was of 8 ±1 µs (n = 4). The optimized command appeared to work equally well with other application pipettes: pipettes of diameters from 100 to 120 µm and septa of 3 to 4 µm thick resulted in pulse durations of 26 ± 1 µs and amplitudes of 92 ± 1%.

By this procedure it was also possible to produce square pulses of 100 µs. After applying the second round of optimized voltage command of 10V amplitude, a 97 ± 3 µs pulse, with 10 µs rise time and 12 µs decay time was obtained (Figure 9). It exchanged 95 ± 2% of the solution. The standard deviation of the pulse duration and the exchanged amplitude measured at successive trials were 3.4 µs and 2.0 %. Three other pipettes showed similar results: 97 ± 2 µs duration, 96 ± 2% of exchange.

## Discussion

The general aim of this study was to find an experimental strategy for producing the short exchange pulses required to separate binding from gating in fast LGIC [6]. Binding of the agonist leads to the opening of the channel. Both processes are tightly coupled: the probability of opening increases with the binding of the agonist molecule and the closing of the pore increases the probability of releasing the agonist. Because both binding and gating kinetics contribute to the increase in macroscopic currents after applying the agonist, it is difficult to tell one contribution from the other. Finding an experimental strategy that allows studying agonist binding separately from channel gating would greatly benefit structure function studies of that LGIC. A possible strategy to resolve the kinetics can be developed by noting that there is a brief period of time at the beginning of the agonist application when there is little gating, simply because there was not enough time for the bound receptors to open. This period lasts a fraction of the gating time constant of LGIC. By restricting the application of the agonist to this early fraction of time, binding occurs nearly without the interference of the gating process. A very fast application makes binding no longer limited by the velocity of the application. Binding is still diffusion limited and limited by the rate of interaction with the binding site once the ligand is present. The shorter duration of the pulse can be counteracted by using a higher concentration of the agonist that results in an increased binding rate. As long as the conformational changes leading to gating are substantially slower than binding period (which is limited to the time where the agonist is present) the separation between binding and gating would be significant. In this way the kinetics of binding could be determined by the measurements of macroscopic currents [3], [28], [29]. The methodology here presented can be used to achieve ca. 20 µs pulses which are sufficient to introduce the agonist to LGIC before their gating occurs.

### Comparison with Other Devices

The flow velocity in our experiments is 40 times bigger than that used in commercial or state of the art devices. Because our system isolates the pressurized gas from the solutions being injected it avoids the formation of bubbles that might collide and break the patch. We also carefully removed air bubbles and filtered solutions of contaminating particles. With this system we obtained flow rates between 100 and 400 cm/s. Higher velocities may be possible but may compromise the stability of the measurement [16], [17] by a grater dynamic pressure on the patch membrane. We thus maintain the dynamic pressure below the rupture limit which is ca. 0.08 bar for a 2 micrometers patch pipette [30], leading to a safe limit of 400 cm/s fluid velocity.

We did not measure the actual flow rate of the solution reaching the measuring tip. For streamline flow in a cylindrical tube, a paraboloidal velocity profile would be expected; the situation here is more complex since two flowing streams are separated by a thin septum and the plume is ejected into the surrounding bath. We therefore presented an estimate based on the volume flow rate and the perfusion tip dimensions.

A high velocity step of the moving perfusion tip might influence the effective width of the interface in the case that the translational velocity is higher than the perfusion velocity. It can even disrupt the interface in the case of a Reynolds number in the turbulent regime (>4000). This was not the case in present experiments. We estimated that the translation velocity of the perfusion tip was at most 16 cm/s for a 10V step. This velocity is 25 times slower than the estimated fluid velocity. Furthermore, even at the high velocities used in this work, the Reynolds number is 400, corresponding to a laminar regime (<2300). Therefore, we do not expect much effect of the piezo translation on the interface width.

High flow rate would have an effect on the membrane tension that would activate mechanosensitive channels. This effect would show up as an increased leakage current. As it is likely that the flow rate would change at different distances from the interface, it is important to perform control experiments with external solution at both streams. Those control experiments would detect if there is an effect of the flow rate gradient over mechanosensitive channels. Furthermore, experiments at different flow rates are necessary to address the possibility of a modulation of the ligand gated receptor by membrane tension.

We found that the width of the interface increases with the width of the partition between the two steams. This effect was more pronounced at high flow velocities because the solution from the bulk is being dragged into the interface especially when the septum is relatively thick. This causes the interface between the streams to become contaminated with solution from the bulk. This fact was made evident by using streams with lower concentrations than that in the bulk solution. Fortunately, this contamination could be alleviated by diminishing the thickness of the septum.

The velocity of the interface movement in our experiments reached 15 cm/s, 4 times faster than the state of the art (3). We also increased the bandwidth to 100 kHz. We used a small excursion high frequency piezo and we drive it with a custom made, 100 kHz, 30 A piezo amplifier. We measured the transfer function of this whole system and after applying simple algebra (Eq. 2) we obtained the time course of the voltage signal to the piezo actuator that produces a pulsed movement of the solution interface.

The combination of the enhancements discussed above made it possible to generate a huge reduction in the duration of the pulse: 25 µs pulses with rise and fall times of 8 µs. In comparison previous arrangements which produced 200 µs pulses with 80 µs rise and fall times using the black box approach on a 10 kHz piezo actuator, with 6 cm/s flow velocity and with a septum 10 µm thick.

The response of the piezo alone shared some characteristics with the response of the whole solution switching system: both lasted about 10 ms and the initial oscillations have a period of 18 µs. However, it is clearly insufficient to predict the response of the whole system. Perfusion, wobbling of the interface and vibrations of the application pipette introduce substantial complexity to the transfer function, stressing the adequacy of measuring directly the movement of the interface in order to optimize its movement. An alternative method to get sub-millisecond pulses [17] has been recently proposed. This consists in finishing the pulse not by returning to the stream where the pipette was before, but by reaching a third stream without changing the direction of the movement. By using PDMS microfluidic technology it is possible to build triple stream switchers that have a middle channel with an exit port width of 10 µm and a septum width of 10 µm. Using a stepper motor that achieves a velocity of 3 cm/s and a flow velocity of 25 cm/s, pulses lasting 400 µs and rise times of 100 µs were obtained.

An option to the use of piezoelectric actuators on parallel streams consists of using solenoid valves as the switching mechanism: the valve turns off one of a pair of colliding streams. In this way 1.0 ms pulses with a rise time of 25 µs [31] were obtained using a flow velocity of 10 cm/s, or much higher flow velocities [32]. The uncertainty on the exact moment the valve is activated that can be reduced by measuring the Plunger Signal [33], which allows determination of that moment.

Our results were obtained on an interface of only 0.36 µm width. Considering that the transverse motion of the stream is 16 cm/s, the moving interface should cross the position of the pipette in just 2 µs. The observed rise time of 8 µs needed for the concentration to change from 10 to 90% was four times slower. Although this value was obtained after correcting for the measured frequency response of the patch clamp amplifier, it might still be limited by the bandwidth of the patch clamp amplifier. In a similar way, previous results on an interface width of 700 nm showed a rise time of 80 µs when the moving interface was calculated to cross in only 14 µs. Aside from the limited bandwidth of the measurements, these discrepancies may arise from diffusion through the unstirred layer that sits against the patch pipette tip. Sachs examined these phenomena using finite element models [24]. His finding was that the rise time depended on the flow velocity of the streams with a power law relationship. When his model is applied to motion of an interface at a flow velocity of 10 cm/s and 100 cm/s, the predicted rise times are 25 and 1.0 µs respectively. To the best of our knowledge, only one set of results reach this maximal performance [31]. The remaining data available today are at best four times slower than this model’s predictions. Further theoretical calculations, wider bandwidth of the exchange measurements and actual measurements of the velocities at the vicinity of the patch pipette are clearly needed to understand these discrepancies.

### Ultra Short Pulses are a Powerful Experimental Tool

The very short pulses here reported would be a powerful tool for the experimental study of the molecular events that occur between the binding of the agonist and the opening of the channel. Pulses lasting 200 microseconds on rat-P2X2 receptors showed a time delay between the application of the agonist and the opening of the channel [3]. This delay was indicative of the presence of an intermediate state between the fully bound closed state and the open state. This state was analogous to a pre-opening state nicknamed flip state found in glycine [34], [35] and ACh receptors [36], and was suggested by the kinetic analysis of single channel recordings. It would in principle be possible to use a 25 µs pulse for detecting the signature of the flip state even in the fastest ligand gated receptors, such as nicotinic ACh or AMPA receptors [7], [37].

In addition, these pulses should be useful to mimic the chemical synapse in the nervous system [38], where the vesicular release mechanism produces a very brief pulse of neurotransmitter leading to a very high transient concentration at the postsynaptic receptors [39]. More than 50% of the neurotransmitter that hits the receptor arrives in 20–50 microseconds. Therefore, to properly understand the response of synaptic receptors it would be necessary to measure the response to pulses of this duration [38], [40], [41]. The effects of potential therapeutic compounds or disease-causing mutations on LGIC currents can be obscured if synaptic conditions are approximated with longer neurotransmitter applications, particularly for fast gating LGIC. The use of very short agonist pulses could help to manifest otherwise subtle differences or effects.
